# Dietary Intervention with Blackcurrant Pomace Protects Rats from Testicular Oxidative Stress Induced by Exposition to Biodiesel Exhaust

**DOI:** 10.3390/antiox11081562

**Published:** 2022-08-12

**Authors:** Michał Oczkowski, Jacek Wilczak, Katarzyna Dziendzikowska, Johan Øvrevik, Oddvar Myhre, Anna Lankoff, Marcin Kruszewski, Joanna Gromadzka-Ostrowska

**Affiliations:** 1 Department of Dietetics, Institute of Human Nutrition Sciences, Warsaw University of Life Sciences (WULS-SGGW), 159c Nowoursynowska Str., 02-776 Warsaw, Poland; 2 Department of Physiological Sciences, Institute of Veterinary Medicine, Warsaw University of Life Sciences (WULS-SGGW), 159 Nowoursynowska Str., 02-776 Warsaw, Poland; 3 Department of Environmental Health, Norwegian Institute of Public Health, Lovisenberggata 8 Str., 0456 Oslo, Norway; 4 Department of Biosciences, Faculty of Mathematics and Natural Sciences, University of Oslo, Blindernveien 31 Str., 0371 Oslo, Norway; 5 Centre for Radiobiology and Biological Dosimetry, Institute of Nuclear Chemistry and Technology, 16 Dorodna Str., 03-195 Warsaw, Poland; 6 Division of Medical Biology, Institute of Biology, Jan Kochanowski University, 7 Uniwersytecka Str., 25-406 Kielce, Poland; 7 Department of Molecular Biology and Translational Research, Institute of Rural Health, 7 Jaczewskiego Str., 20-090 Lublin, Poland

**Keywords:** testis, biodiesel exhaust emission, 1st and 2nd generation biodiesel fuels, blackcurrant pomace, oxidative stress

## Abstract

The exposure to diesel exhaust emissions (DEE) contributes to negative health outcomes and premature mortality. At the same time, the health effects of the exposure to biodiesel exhaust emission are still in scientific debate. The aim of presented study was to investigate in an animal study the effects of exposure to DEE from two types of biodiesel fuels, 1st generation B7 biodiesel containing 7% of fatty acid methyl esters (FAME) or 2nd generation biodiesel (SHB20) containing 7% of FAME and 13% of hydrotreated vegetable oil (HVO), on the oxidative stress in testes and possible protective effects of dietary intervention with blackcurrant pomace (BC). Adult Fisher344/DuCrl rats were exposed by inhalation (6 h/day, 5 days/week for 4 weeks) to 2% of DEE from B7 or SHB20 fuel mixed with air. The animals from B7 (*n* = 14) and SHB20 (*n* = 14) groups subjected to filtered by a diesel particulate filter (DPF) or unfiltered DEE were maintained on standard feed. The rats from B7+BC (*n* = 12) or SHB20+BC (*n* = 12), exposed to DEE in the same way, were fed with feed supplemented containing 2% (m/m) of BC. The exposure to exhaust emissions from 1st and 2nd generation biodiesel resulted in induction of oxidative stress in the testes. Higher concentration of the oxidative stress markers thiobarbituric acid-reactive substances (TBARS), lipid hydroperoxides (LOOHs), 25-dihydroxycholesterols (25(OH)2Ch), and 7-ketocholesterol (7-KCh) level), as well as decreased level of antioxidant defense systems such as reduced glutathione (GSH), GSH/GSSG ratio, and increased level of oxidized glutathione (GSSG)) were found. Dietary intervention reduced the concentration of TBARS, 7-KCh, LOOHs, and the GSSG level, and elevated the GSH level in testes. In conclusion, DEE-induced oxidative stress in the testes was related to the biodiesel feedstock and the application of DPF. The SHB20 DEE without DPF technology exerted the most pronounced toxic effects. Dietary intervention with BC in rats exposed to DEE reduced oxidative stress in testes and improved antioxidative defense parameters, however the redox balance in the testes was not completely restored.

## 1. Introduction

Over the last few decades, several studies have emphasized adverse health effects of diesel exhaust emissions (DEE), manifested, among others, by reduced lung function, irritation symptoms, inflammatory responses, cardiovascular effects, and premature deaths [1,2,3,4,5,6]. The health effects of DEE result mainly from emission of gaseous chemicals, such as nitrogen oxides (NOx), hydrocarbons (HC), carbon monoxide (CO), soot, and emission of particulate matter (PM) [7]. Diesel PM is mostly composed of elemental carbon core and substances adsorbed on its surface, such as polycyclic aromatic hydrocarbons (PAH), PAH-derivatives, and inorganic compounds (metals, ions, inorganic acids, salts). Both gaseous and particulate components of DEE induce cellular oxidative stress, causing DNA oxidation and single strand break formation, as well as stimulate release of inflammatory cytokines (such as interleukin (IL)-1α and -β and tumor necrosis factor (TNF)-α, as well as IL-8), leading to higher risk of autoimmune reactions [8,9,10].

The exposure of animals to DEE has previously been shown to increase morphological sperm abnormalities, induce ultrastructural changes in Leydig cells, and reduce the level of luteinizing hormone (LH) [11]. Further in vivo studies demonstrated that DEE increased testicular concentration of testosterone and induced degeneration of tubules [12]. In another study, it was found that chronic exposure to DEE impaired the fertility of male mice, decreased the sperm count/motility, and disrupted the spermatogenesis [13]. While the mechanisms underlying the adverse reproductive and developmental effects of DEE from combustion of diesel or biodiesel blends are not fully understood, the promotion of oxidative stress and inflammation have been shown to play an important role in the reproductive toxicity [14].

As significant reduction in DEE exposure due to reduction of diesel oil consumption is currently unlikely, lifestyle changes might be the option to reduce reproductive toxicity, such as intake of dietary natural phytochemicals with established anti-inflammatory and antioxidant properties. Among dietary natural phytochemicals, blackcurrant fruits (Ribes nigrum) (BC) exhibit strong antioxidant, anti-inflammatory, antimicrobial, antitumor, and immunomodulatory properties due to the presence of many polyphenolic compounds, such as flavonoids and anthocyanins [15,16,17,18,19,20,21]. Due to their ROS-scavenging, anti-inflammatory, and metal-chelating abilities blackcurrant fruits have been intensively studied during recent decades [22]. However, to the best of our knowledge there are no data describing a potential protective role of blackcurrant fruits against DEE-induced reproductive toxicity in vivo. Therefore, the present study was conducted to address this question with two main objectives: to determine whether 28-day exposure to DEE from combustion of the 1st and 2nd generation biofuels disrupt the testicular oxidative/anti-oxidative balance and to investigate whether a dietary intervention with blackcurrant pomace can attenuate the DEE-induced testicular oxidative stress in Fisher344/DuCrl rats.

## 2. Materials and Methods

### 2.1. Reagents and Chemicals

The reagents and chemicals were purchased from Sigma-Aldrich (St. Louis, MO, USA), unless otherwise indicated. The list of reagents and chemicals necessary for animal experiment and biochemical analyses were enclosed in Supplementary Materials.

### 2.2. Animal Study

**Figure 1:**
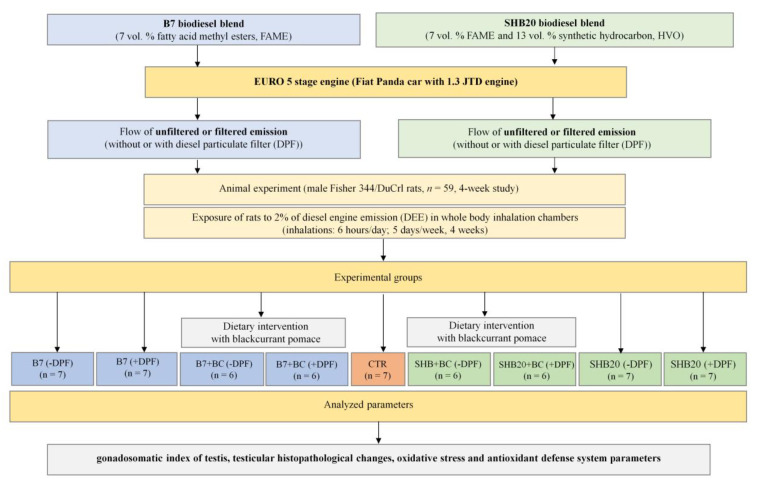
Experimental design of the in vivo experiments. B7—1st generation biofuel; SHB20—2nd generation biofuel; DPF—diesel particulate filter; DEE—diesel engine emissions; BC—blackcurrant pomace.

All procedures were approved by the Third Local Animal Care and Use Committee in Warsaw, Poland (Certificate of Approval No WAW3/04/2014) according to Polish and UE standards and law regulations, as well as in line with 3R rules. Healthy adult male Fisher344/DuCrl rats (306.9 ± 1.9 g at the beginning of experiment, *n* = 59) were obtained from Charles River Laboratories, Inc. (Schulzfeld, Germany). The rats acclimatized to animal house conditions in the Institute of Veterinary Medicine (WULS-SGGW) for 1 week (22 ± 1 °C, 50 ± 5% relative humidity, 12 h light–dark cycle). The animals were fed with AIN-93M pellets for laboratory rats (ZooLab, Sędziszów, Poland) and had free access to water. After the acclimatization period, the rats were randomly assigned into 9 groups, including 8 experimental groups and 1 control group, according to the scheme presented in Figure 1.

### 2.3. Exposure of Animals to DEE

The exposure of rats to DEE and detailed chemical exhaust analysis have been previously described by our group [23,24,25,26]. Briefly, DEE were generated from a Fiat Panda 1.3 JDT (2014) with a Euro 5 engine (Common Rail 3rd generation injection system, 1248 cm^3^, max. power 75 bhp, max. torque 190 Nm). Rats were exposed in whole body inhalation chambers to DEE from two biodiesel blends (B7—the 1st generation biofuel containing 7% *v/v* fatty acid methyl esters (FAME) or SHB20—the 2nd generation biofuel containing 7% *v/v* FAME and 13% *v/v* synthetic hydrotreated vegetable oils (HVO)) diluted to 2% with air, according to the scheme presented at Figure 1.

The inhalation procedure was described in detail in our previously published papers [23,24,25,27] and was also illustrated in Figure S2 in Supplementary Materials. Estimated concentrations of substances in the air inside the test chambers (Tables S6 and S7 in Supplementary Materials) were described in our previously published paper [23]. For inhalation (6 h/day; 5 days a week for 4 weeks, according to OECD Guideline for the Testing of Chemicals No 412 [28]), the individual cages with rats were placed in two separate inhalation chambers equipped with a rack for cages). A modification of the car engine allowed to supply an unfiltered or filtered DEE using diesel particulate filter (DPF) technology. Inhalation was performed separately for each type of biodiesel blend. Animals from four experimental groups (B7 (+DPF), SHB20 (+DPF), B7 (-DPF), SHB20 (-DPF)), exposed to filtered or unfiltered DEE, were maintained on standard feed without blackcurrant pomace (BC). Another four experimental groups (B7+BC (+DPF), SHB20+BC (+DPF), B7+BC (-DPF), SHB20+BC (-DPF)) exposed in the same condition (Figure 1) were maintained on the same feed supplemented with BC (20 g/kg feed). The animals were given feed and water ad libitum. Chemical analysis of animal feed was performed in Merieux NutriScience Silliker Laboratory (Warsaw, Poland) and the results are presented in Table S1 (Supplementary Materials). Chemical analysis of selected flavonoids and phenolic acids in blackcurrant pomace confirmed that more than 93% (*w/w*) of analyzed compounds were anthocyanins. The characteristic of phenolics in experimental feed is presented in Table S2 (Supplementary Materials). Individual feed consumption was monitored once a day and body weight gain of rats were determined weekly.

After the experiment (4 weeks), rats were anesthetized with isoflurane (Aerrane Isofluranum, Baxter, Deerfield, IL, USA) and bled by heart puncture. Next, both testes were dissected, washed with ice-cold PBS, weighted, and frozen in liquid nitrogen. The tissues were stored in −80 °C for biochemical analysis. Testicular tissue was homogenized in 5–10 mL cold buffer (50 mM potassium phosphate, pH 7.5 with 1 mM EDTA) per gram of tissue and then centrifuged at 10,000× *g* for 15 min at 4 °C.

### 2.4. Gonadosomatic Index

Gonadosomatic index (GSI, %) was calculated separately for each rat according to the following formula: GSI = (mass of both testes (g)/final body weight (g)) × 100% [29].

### 2.5. Histological Assessment of Testis

The collected testicular samples were fixed in 10% buffered formaldehyde for 24 h and embedded in paraffin. Four-micron sections were cut from paraffin blocks, fixed on microscope glass slides, stained with hematoxylin and eosin, and evaluated by a veterinary pathologist.

### 2.6. Oxidative Stress Parameters in Testis

The level of testicular lipid peroxidation was evaluated based on formation of malondialdehyde (MDA) by measuring thiobarbituric acid-reactive species (TBARS) as described by Ohkawa et al. [30] with some modifications. The TBARs concentration was calculated from a standard curve using 1,1,3,3-tetramethoxypropane, which gives MDA upon hydrolysis during the assay. The concentration of lipid hydroperoxides in testicular homogenates was analyzed by the method of Yagi [31]. The absorbance of samples was read at 665 nm. The concentration of lipid hydroperoxides was calculated from a standard curve using cumene hydroperoxide. The concentrations of 25-dihydroxycholesterol (25(OH)2Ch) and 7-ketocholesterol (7-KCh), the two major cholesterol oxidation by-products, were assessed in testicular homogenates using the high-performance liquid chromatography (HPLC-UV) method according to Suchecka et al. [32]. Analyses of lipid peroxidation, lipid hydroperoxides, and oxysterols were assessed in technical duplicates.

### 2.7. Anti-Oxidative Defense System Parameters in Testis

The level of total antioxidant status (TAS) and activity of superoxide dismutase (SOD), glutathione peroxidase (GPx), and reductase (GR) in the testis homogenates were determined spectrophotometrically using Randox reagents (Randox Laboratories Ltd., Crumlin, Co. Antrim, UK), cat no: NX2332, SD125, RS505, GR2368, respectively, according to the manufacturer’s protocols. Reduced glutathione (GSH) and its oxidized form glutathione disulfide (GSSG) were determined using HPLC method [33,34] according to the procedures described in [35]. The analysis of TAS level and enzymes activities were performed in technical duplicates.

### 2.8. Statistical Analysis

Statistical analysis was conducted using Statistica software version 13.3 [36]. All data were analyzed using two-way analysis of variance (ANOVA) followed by post hoc Duncan’s test separately for animals exposed to unfiltered and filtered DEE. The comparison of data from experimental groups versus the control group was performed using one-way analysis of variance (ANOVA) followed by post hoc Duncan’s test. Statistical significance was set at *p* < 0.05. All the results were expressed as mean ± SEM (Standard Error of Mean). The figures were performed in GraphPad Prism version 9.2.0 (332) for Windows, GraphPad Software, San Diego, CA, USA [37].

## 3. Results

### 3.1. Animal Observation, Weight Changes, Gonadosomatic Index, and Microscopic Evaluation of Rat Testis

All rats appeared to be in a good health condition without any negative behavioral symptoms throughout the experimental period. Moreover, no exposure-related mortality was noted. The initial animals body weights did not significantly differ between experimental groups (data not shown). Exposure to DEE and dietary intervention with BC pomace revealed lower weekly food intake of rats from SHB20+BC exposed to unfiltered DEE compared with B7+BC and CTR group (Figure S1A,B). Higher average daily intake of phenolic compounds was observed in rats from B7+BC exposed to DEE with or without DPF-treatment compared to animals from SHB20+BC group (Table S3, Supplementary Materials).

**Figure 2:**
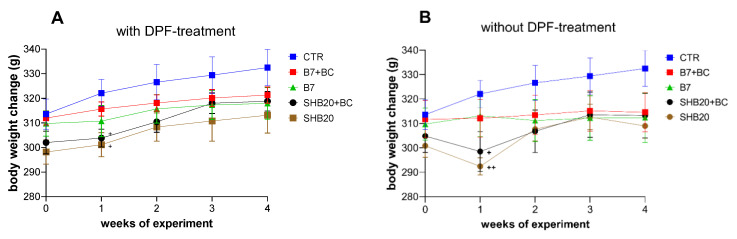
Body weight change of rats exposed to DEE from B7 or SHB20 biofuels with (**A**) or without (**B**) DPF-treatment and with or without blackcurrant pomace supplementation (BC) during the experiment. CTR—control group; B7—1st generation biofuel; SHB20—2nd generation biofuel; BC—blackcurrant pomace; DPF—diesel particulate filter. Data are expressed as mean ± SEM; ^+^—Statistically significant difference from the control group (CTR), ^+^ *p* < 0.05; ^++^ *p* < 0.01, one-way ANOVA with Duncan’s post hoc test.

The mean initial body weight of rats did not significantly differ between control and experimental groups of rats exposed to DEE with or without DPF-treatment. However, the animals from CTR group had a higher initial body weight, the results of one-way ANOVA at each time point revealed that only after first week of the experiment significantly lower body weight of animals from SHB20 and SHB20+BC groups exposed to DEE with or without DPF-treatment were noted, as compared to control group (Figure 2A,B). Dietary intervention with BC did not affect the weight of rats significantly.

**Figure 3:**
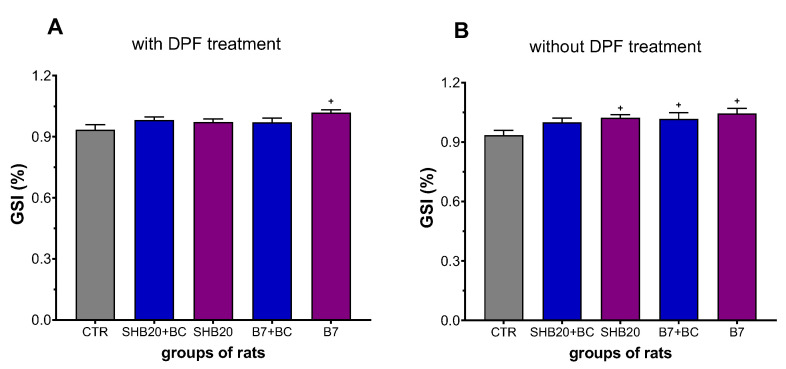
Gonadosomatic index (GSI) of testes of rats exposed to DEE from B7 or SHB20 biofuels without (**A**) or with (**B**) DPF-treatment and with or without blackcurrant pomace supplementation (BC) for 28 days. CTR—control group; B7—1st generation biofuel; SHB20—2nd generation biofuel; BC—blackcurrant pomace; DPF—diesel particulate filter; ^+^ denotes statistically important difference vs. CTR group, *p* < 0.05. Data are expressed as mean ± SEM.

The effect of DEE exposure on the testis gonadosomatic index (GSI) is shown in Figure 3A,B). Analysis of variance (ANOVA) with Duncan’s post hoc test revealed a significant increase of GSI in rats from B7 (with DPF-treatment) groups (Figure 3A), as well as in animals from B7 (with or without BC) and SHB20 groups without DPF-treatment (Figure 3B), as compared to the corresponding control group (*p* < 0.05). The results showed no effect of BC supplementation on male GSI.

**Figure 4:**
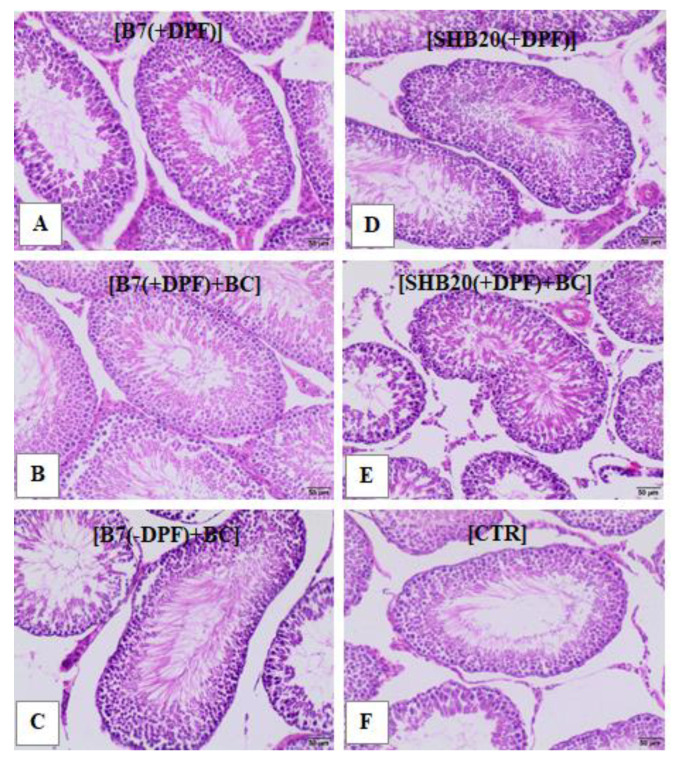
Representative microphotographs of hematoxylin-eosin-stained cross sections of testes of rats exposed to DEE from B7 or SHB20 biofuels with or without DPF-treatment and with or without blackcurrant pomace supplementation (BC) for 28 days. Testes of rats exposed to (**A**) filtered DEE from B7 biodiesel blend, (**B**) filtered DEE from B7 biodiesel blend and fed with feed with BC pomace, (**C**) unfiltered DEE from B7 biodiesel blend and fed with BC pomace, (**D**) filtered DEE from SHB20 biodiesel blend, (**E**) filtered DEE from SHB20 biodiesel blend and fed with feed with BC pomace, and (**F**) testes of control rats. B7—1st generation biofuel; SHB20—2nd generation biofuel; BC—blackcurrant pomace; DPF—diesel particulate filter; CTR—control group. Images were captured at 400× magnification; scale bars correspond to 50 μm.

In all groups, histological analysis of testicular sections revealed normal architecture of the seminiferous tubules, normal interstitial compartment with Leydig cells, and regular seminiferous epithelium containing the germ cells in all stages of differentiation and normal tubular lumen (Figure 4).

### 3.2. Oxidative Stress Markers

**Figure 5:**
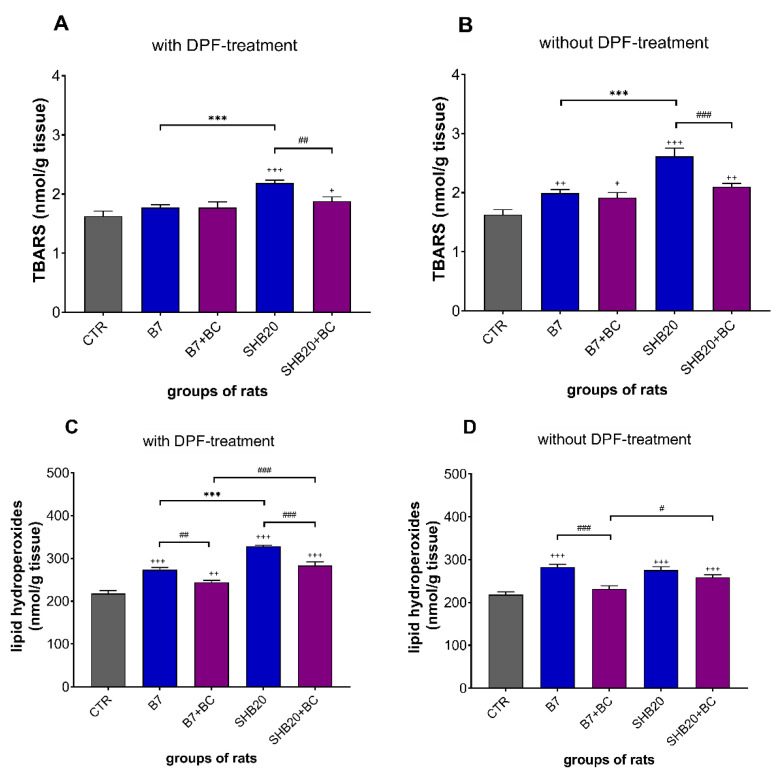
The concentration of thiobarbituric acid reactive substances (TBARS) (**A**,**B**) and lipid hydroperoxides (LOOHs) (**C**,**D**) in the testes of rats exposed to DEE from B7 or SHB20 biofuels with and without DPF-treatment and with or without blackcurrant pomace supplementation (BC) for 28 days. Data are expressed as mean ± SEM. ^#^—significant difference between normal and blackcurrant pomace supplemented diet, ^#^ *p* < 0.05; ^##^ *p* < 0.01; ^###^ *p* < 0.001; *—Statistically significant difference between B7 and SHB20 DEE, *** *p* < 0.001; ^+^—Statistically significant difference from the control group (CTR), ^+^ *p* < 0.05; ^++^ *p* < 0.01; ^+++^ *p* < 0.001, one-way ANOVA with Duncan’s post hoc test.

As illustrated in Figure 5A,B, the exposure of rats to SHB20 DEE with DPF-treatment and B7 or SHB20 DEE without DPF-treatment significantly increased the concentration of TBARS in the testes compared with the control group. There was a significant difference in TBARS concentration between B7 and SHB20 DEE groups, with higher concentration in the testes of rats exposed to SHB20 DEE with and without DPF-treatment. Dietary intervention with BC significantly reduced increase of concentration of TBARS in the testes of rats exposed to SHB20 DEE without or with DPF-treatment. Figure 5C,D show that in the testes of rats exposed to B7 or SHB20 DEE without and with DPF-treatment, with exception of B7(-DPF)+BC group, the concentration of lipid hydroperoxides (LOOHs) significantly increased, compared with the control group. There was a significant difference in the concentration of LOOHs in the testes between B7 and SHB20 DEE groups, with higher concentration in rats exposed to SHB20 DEE with DPF. Dietary intervention significantly ameliorated the increase in concentration of LOOHs in the testes of rats exposed to B7 DEE without DPF-treatment and in rats exposed to B7 and SHB20 DEE with DPF-treatment.

**Figure 6:**
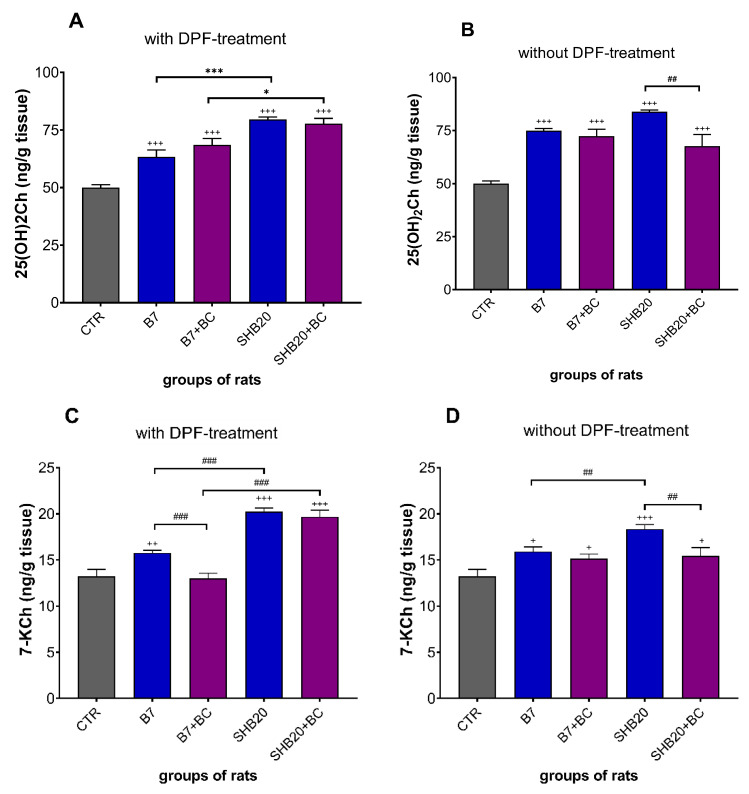
The concentration of 25-dihydroxycholesterol (25(OH)2Ch) (**A**,**B**) and 7-ketocholesterol (7-KCh) (**C**,**D**) in the testes of rats exposed to DEE from B7 and SHB20 biofuels with and without DPF-treatment and with or without blackcurrant pomace supplementation (BC) for 28 days. Data are expressed as mean ± SEM. ^#^—Statistically significant difference between normal and blackcurrant pomace supplemented diet, ^##^ *p* < 0.01; ^###^ *p* < 0.001; *—Statistically significant difference between B7 and SHB20 DEE, * *p* < 0.05; *** *p* < 0.001; ^+^—Statistically significant difference from the control group (CTR), ^+^ *p* < 0.05; ^++^ *p* < 0.01; ^+++^ *p* < 0.001, one-way ANOVA with Duncan’s post hoc test.

As presented in Figure 6A,B, the exposure of rats to B7 or SHB20 DEE with and without DPF-treatment significantly enhanced the concentration of oxidized cholesterol metabolite (25(OH)2Ch), versus the control group. There was a significant difference in the concentration of 25(OH)2Ch between B7 and SHB20 DEE groups, with higher concentration in the testes of rats exposed to DEE from SHB20 biodiesel fuel with DPF-treatment. The latter effects of DEE were prevented by dietary intervention with BC in rats exposed to SHB20 DEE without DPF-treatment.

The results of 7-KCh concentration in the testis, presented in Figure 6C,D, showed that the rats exposed to B7 or SHB20 DEE with or without DPF-treatment revealed significantly increased concentrations of this oxysterol compared to CTR group, except the animals from B7+BC group exposed to DEE with DPF-treatment. Furthermore, the animals exposed to DEE from SHB20 biodiesel fuel with or without DPF-treatment presented higher concentration of 7-KCh in testis than the rats exposed to DEE from B7 biofuel. The effects of DEE were inhibited by dietary intervention with BC in rats exposed to B7 DEE with DPF-treatment and in animals exposed to SHB20 DEE without DPF-treatment.

### 3.3. Antioxidant Defense Markers

**Figure 7:**
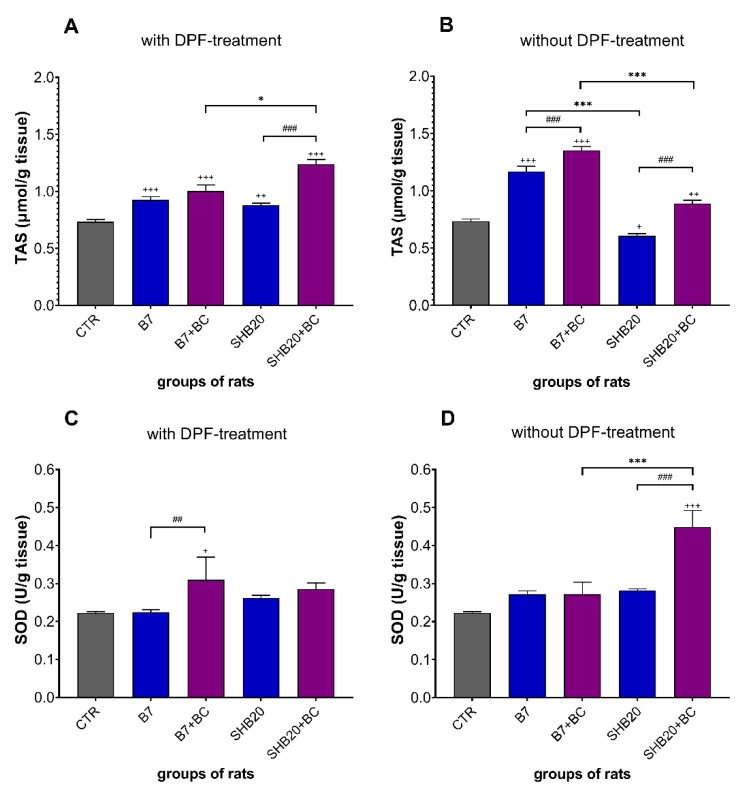
The total antioxidant status (TAS) (**A**,**B**) and superoxide dismutase (SOD) activity (**C**,**D**) in the testes of rats exposed to DEE from B7 and SHB20 biofuels with or without DPF-treatment and with or without blackcurrant pomace supplementation (BC) for 28 days. Data are expressed as mean ± SEM. ^#^—Statistically significant difference between normal and blackcurrant pomace supplemented diet, ^##^ *p* < 0.01; ^###^ *p* < 0.001; *—Statistically significant difference between B7 and SHB20 DEE, * *p* < 0.05; *** *p* < 0.001; ^+^—Statistically significant difference from the control group (CTR), ^+^ *p* < 0.05; ^++^ *p* < 0.01; ^+++^ *p* < 0.001, one-way ANOVA with Duncan’s post hoc test.

**Figure 8:**
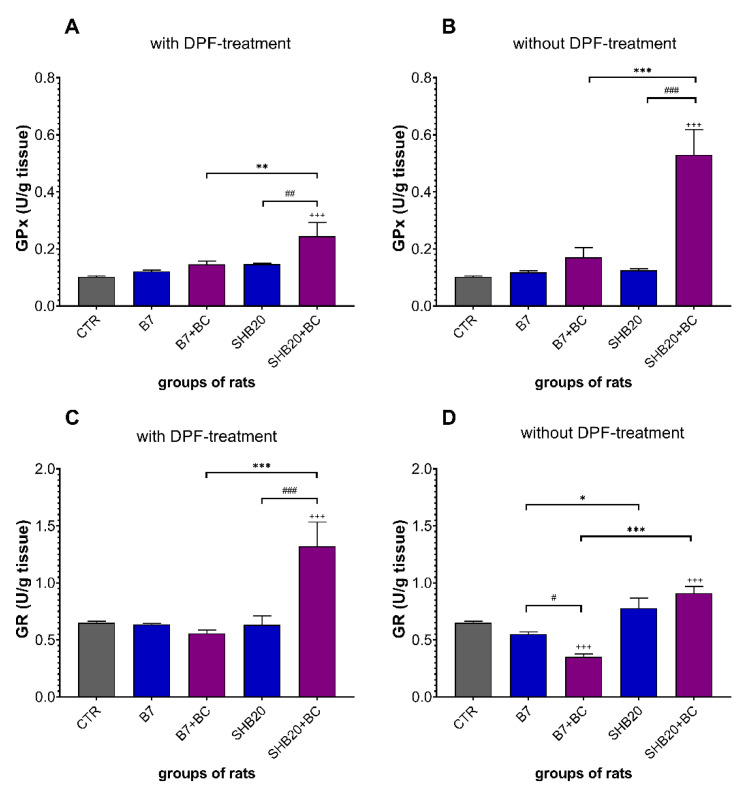
The activity of glutathione peroxidase (GPx) (**A**,**B**) and glutathione reductase (GR) (**C**,**D**) in the testes of rats exposed to DEE from B7 or SHB20 biofuels with or without DPF-treatment and with or without blackcurrant pomace supplementation (BC) for 28 days. Data are expressed as mean ± SEM. ^#^—Statistically significant difference between normal and blackcurrant pomace supplemented diet, ^##^ *p* < 0.01; ^###^ *p <* 0.001; *—Statistically significant difference between B7 and SHB20 DEE, * *p* < 0.05; ** *p* < 0.01; *** *p* < 0.001; ^+^—Statistically significant difference from the control group (CTR), ^+++^ *p* < 0.001, one-way ANOVA with Duncan’s post hoc test.

**Figure 9:**
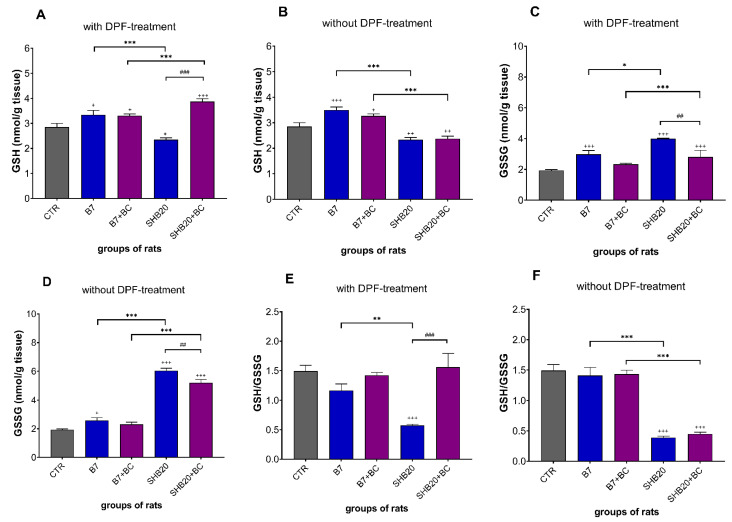
The concentration of reduced form of glutathione (GSH) (**A**,**B**), oxidized form of glutathione (GSSG) (**C**,**D**) and GSH to GSSG ratio (**E**,**F**) in the testes of rats exposed to DEE from B7 and SHB20 biofuels with and without DPF-treatment and with or without blackcurrant pomace supplementation (BC) for 28 days. Data are expressed as mean ± SEM. ^#^—Statistically significant difference between normal and blackcurrant pomace supplemented diet, ^##^ *p* < 0.01; ^###^ *p* < 0.001); *—Statistically significant difference between B7 and SHB20 DEE, * *p* < 0.05; ** *p* < 0.01; *** *p* < 0.001); ^+^—Statistically significant difference from the control group (CTR), ^+^ *p* < 0.05; ^++^ *p* < 0.01; ^+++^ *p* < 0.001, one-way ANOVA with Duncan’s post hoc test.

The effects of dietary intervention with blackcurrant pomace in rats exposed to B7 and SHB20 DEE without and with DPF-treatment on antioxidant defense markers are shown in Figure 7, Figure 8 and Figure 9. The exposure of rats to B7 or SHB20 DEE without and with DPF-treatment, with exception of SHB20(-DPF) group, increased the total antioxidant potential (TAS) versus controls (Figure 7A,B). There was a significant difference in the TAS between B7 and SHB20 DEE groups, with higher level in the testes of rats exposed to B7 DEE without DPF-treatment. Dietary intervention significantly increased the TAS in the testes of rats exposed to B7 and SHB20 DEE without and with DPF-treatment.

Figure 7C,D show that the superoxide dismutase (SOD) activity was significantly higher only in the testes of rats exposed to SHB20 DEE without DPF-treatment and with dietary intervention, and in the testes of rats exposed to B7 DEE with DPF-treatment and with BC dietary intervention, compared to the corresponding groups without dietary intervention.

As presented in Figure 8A,B, there was no difference in the activity of glutathione reductase (GPx) in the testes between control rats and rats exposed to B7 or SHB20 DEE without and with DPF-treatment. Dietary intervention with BC significantly increased the activity of GPx in the testes of rats exposed to SHB20 DEE without and with DPF, compared to the corresponding groups without dietary intervention.

As illustrated in Figure 8C,D, no changes of the activity of glutathione reductase (GPx) in the testes were observed between rats exposed to B7 and SHB20 DEE without and with DPF-treatment and the control rats. Dietary intervention with BC markedly increased the activity of GR in the testes of rats exposed to SHB20 DEE with DPF-treatment but reduced the activity of GR in the testes of rats exposed to B7 DEE without DPF-treatment, compared to the corresponding groups without dietary intervention.

Figure 9A,B shows that the concentration of reduced form of glutathione (GSH) in testes was significantly higher in groups of rats exposed to B7 DEE regardless the DPF-treatment and in rats from SHB20+BC group inhaled with filtered DEE. However, markedly lower level of GSH was demonstrated in both groups of rats exposed to SHB20 DEE (without and with DPF-treatment) as well as in rats from SHB20 group with DPF-treatment, versus controls. Dietary intervention with BC significantly increased the concentration of GSH in the testes of rats exposed to SHB20 DEE with DPF-treatment, as compared to the corresponding group without dietary intervention. In turn, the concentration of oxidized form of glutathione (GSSG) (Figure 9C,D) was significantly higher in rats exposed to B7 and SHB20 DEE without and with DPF-treatment, compared to the control group. Dietary intervention with BC significantly decreased the concentration of GSSG in the testes of rats exposed to SHB20 DEE without and with DPF-treatment, compared to the corresponding groups without dietary intervention.

Figure 9E,F shows a significant reduction in the cellular GSH:GSSG ratio in the testes of rats exposed to SHB20 DEE without or with DPF-treatment, compared to the control group. Dietary intervention prevented a drop in the GSH:GSSG ratio only in the testes of rats exposed to SHB20 DEE with DPF-treatment, compared to the corresponding group without BC.

## 4. Discussion

Our results revealed that even though exposure to DEE from the combustion of both biofuels had no effect on the general health condition and food intake, a significantly lower body weight gain was observed in rats after the first week of inhalation. This result differs from other studies, which revealed no relationship between exposure to DEE and body weight gain in adult animals [12,38]. However, the studies on the effects of in utero DEE exposure showed the reduced body weight of male offspring at postnatal day 90 [39]. A precise mechanism of these effects has not yet been established.

The differences in the body weight gain did not affect the testis morphology. Regardless of the observed increase in relative testicular weight (GSI) in rats exposed to B7 DEE (with or without DPF), as well as in SHB20 and B7+BC (without DPF) groups compared to control animals, the rats from all experimental groups displayed normal morphology of the testes. This result is in line with the observation published by Watanabe and Oonuki [40], who reported no structural malformations in the testes from diesel exhaust-exposed rats. In contrast to these findings, Kisin et al. [11] and Yang et al. [13] reported clustering of the dystrophic seminiferous tubules with arrested spermatogenesis and the presence of degenerating spermatocytes in mice exposed to biodiesel B50. Similarly, Ono et al. [41] and Kubo-Irie et al. [42] showed that exposure to DEE during pregnancy caused harmful effects in the testes of male offspring, manifested as degenerated seminiferous tubules with multinucleated giant cells. The observed discrepancy between our results and conclusions from other publications is likely caused by the differences in the type of fuel, exposure conditions, and animal species. We tested two types of biofuels: B7 biofuel (containing 7% FAME—fatty acid methyl esters with the chemical structure CH_3_(CH2)_n_COOCH_3_) and SHB20 biofuel (containing 7% *v/v* FAME and 13% *v/v* HVO—hydrotreated vegetable oils free of aromatics, oxygen and sulfur with the chemical structure CnH2n+2) [43]. The exposure of rats to the gaseous and particulate emissions from diesel engine for both fuels (2.1–2.2% (*v/v*) of inhaled air was environmentally relevant and was within the range that humans are likely to be exposed to in a city agglomeration. Based on the results presented in Tables S6 and S7 (in Supplementary Materials), higher concentration of unburned hydrocarbons inside the inhalation chamber with DPF-treatment was noted regardless of the type of biodiesel blend. Moreover, the application of DPF resulted in reduction of particulate matter concentration from B7 compared to SHB20 biodiesel emission (by 92% and 91%, respectively). The concentration of polycyclic aromatic hydrocarbons (PAHs) differed depending on the type of biodiesel blend and DPF-treatment. Generally, more than 90% reduction of PAHs concentration was noted inside the chambers with DPF application. However, Kisin et al. [11] tested B50 biofuel containing 50% *v/v* FAME and determined abnormalities in the mice reproductive system after exposure to particulate emissions from diesel engine with a cumulative dose of 60 µg per mouse of total carbon. Likewise, the other authors listed above examined detrimental effects of particulate emissions (1.0 mg/m^3^, from day 2 until day 16 post coitum) from diesel engine on mouse spermatogenesis in offspring. Our assumption is also consistent with the results of our and other experimental studies showing that the type of biofuel and concentration of biodiesel blends may affect the toxicological characteristics of diesel particulate matter due to potential increases of certain toxic compounds in its composition [23,44,45]. Dietary intervention with blackcurrant pomace had no effect on the food intake, body weight gain, gonadosomatic index (GSI) of testes and the architecture of seminiferous tubules of rats exposed to DEE.

Dysfunction of testes is suggested to be associated with induction of oxidative stress. Due to relatively higher level of unsaturated fatty acids in testes than in other organs, male gonads are particularly susceptible to increased oxidative stress induced by, e.g., different environmental factors [46,47]. A well-known symptom of oxidative stress is peroxidation of lipids due to the overproduction of free radicals and peroxides in the intracellular and extracellular environment. We therefore decided to measure malondialdehyde (MDA) as the thiobarbituric acid-reactive-substances (TBARS), which is a critical biomarker in the study of polyunsaturated fatty acids (PUFAs) peroxidation [48,49]. Our results revealed that the exposure of rats to B7 and SHB20 DEE significantly increased the concentration of TBARS, but this effect was the most pronounced in rats treated with SHB20 DEE without DPF-treatment. These results corroborate with results of Liu et al. [50,51], who demonstrated the increased concentration of MDA in testes of rats intratracheally administered with PM2.5 from DEE. The increased level of MDA was also observed in the testes of mice exposed to 3-methyl-4-nitrophenol, a component of diesel exhaust particles [52]. Aside from the TBARS, lipid hydroperoxides (LOOHs) are useful biomarkers of early-stage lipid peroxidation. Lipid hydroperoxides, a very unstable products of reaction of peroxyl radical (ROO^•^) polyunsaturated fatty acids, are considered as one of the most important markers of cellular oxidative stress [53,54]. Our findings showed that the exposure of rats to DEE significantly increased the concentration of LOOHs, with the highest concentration in rats exposed to SHB20 DEE with DPF-treatment. Another reliable marker of oxidative stress in vivo is peroxidation of products of cholesterol metabolism, namely oxysterols [55]. In the present study, we determined the levels of two oxysterols, which are the most common products of reaction between cholesterol and oxygen radicals: 25-dihydroxycholesterol (25(OH)2Ch) and 7-ketocholesterol (7-KCh) [56,57]. 7-ketocholesterol is the most toxic oxysterol within the organism and exerts a strong impact to form ROS in the cells and promote oxiapoptophagy, a cell death process induced by certain oxysterols [56]. We noted that both types of DEEs significantly enhanced the concentration of 25(OH)2Ch and 7-KCh in the testes. DEE from SHB20 biodiesel fuel appeared to be the most potent in increasing of 25(OH)2Ch and 7-KCh concentrations. To the best of our knowledge, the effects of DEE from the combustion of biodiesel fuels on the generation of oxidatively modified forms of cholesterol in the testes has not been evaluated so far, but Rao et al. [58] reported a marked increase in 7-KCh concentration in plasma lipoproteins (VLDL and LDL/IDL) and in the aorta of mice in response to inhaled particulate matter (PM2.5) during chronic air pollution exposure.

Based on the above-mentioned data, our results clearly demonstrate that both B7 and SHB20 DEE induced oxidative stress in the testes of rats, and the effects were higher for SHB20. Moreover, the DPF-treatment reduced several of the effects. The highest toxic effects were observed for SHB20 DEE without DPF technology. We believe that the observed differences in the oxidative toxicity between B7 DEE and SHB20 DEE resulted from the dissimilarity in the composition of the DEE-derived particles. Some studies investigating the exposure of animals to DEE particulate matter indicated that alteration of applied engine load changed the composition and biological reactivity of particles [59,60]. Indeed, the analysis of DEE from the combustion of both biofuels used in this study clearly demonstrated a difference in chemical characteristics of emissions from the two tested fuels [26,61,62]. Though the concentrations of the gaseous components (CO, CO_2_, NO_x_) were approximately in the same range for B7 and SHB20 DEE, the combustion of SHB20 biofuel generated a higher number of ultrafine particles with diameter less than 100 nm (~85%) than the combustion of B7 biofuel (~55%). Compared with larger particles, ultrafine particles penetrate rapidly into various body organs, are cleared more slowly, retain longer after deposition, and have a greater ability to deposit toxic chemicals on the surface due to a larger surface-to-mass ratio [63,64]. In line, the observed higher toxicity of DEE without DPF-treatment, as compared with DPF-treatment, was most likely related to a higher concentration of particulate matter. The efficiency of DPF-treatment was previously confirmed in our FuelHealth project, showing a reduced total mass of particulate matter by approximately 90% [61].

The higher pro-oxidative properties of SHB20 DEE exposure, as compared with B7 DEE, may be attributed to the presence of redox-active transition metals, present only in SHB20-derived particles [62]. The particle-associated metals, such as copper and iron might undergo Fenton reaction, promoting formation of free radicals [65,66]. However, the effects might also be due to more secondary responses arising from activation of cellular redox machinery [63].

Since oxidative stress develops because of an imbalance favoring oxidants, leading to a disrupted redox signaling and molecular damage, we investigated the effects of DEE on antioxidant defense systems [67,68,69], expressed as total antioxidant status (TAS), which describes the dynamic equilibrium between prooxidants and antioxidants [70], and the level/activity of antioxidant enzymes and low molecular weight radical scavenger. In our study, the treatment with DEE from the combustion of both biofuels without and with DPF-treatment significantly increased the level of TAS, with the highest value in the testes of rats exposed to B7 DEE without DPF-treatment. The opposite results were presented by Nemmar et al. [71], who studied the interaction of diesel exhaust particles with human, rat and mouse erythrocytes in vitro and found that TAS was decreased in a dose-dependent manner in rat and mouse erythrocytes but was not affected in human erythrocytes. The observed increase of TAS may, in our opinion, reflect a redox response.

Apart from TAS, some enzymatic antioxidants, such as superoxide dismutase (SOD), glutathione peroxidase (GPx), and glutathione reductase (GR), as well as non-enzymatic antioxidants, such as glutathione (GSH), play important roles in the antioxidative defense system. In the present study, we did not find a statistically significant difference in the activity of SOD, GPx, and GR in testicular homogenates from the exposed groups. Similar observations were already reported for the testicular GPx activity in male rats after treatment with motorcycle exhaust [72]. However, previous studies examining the testicular activity of SOD and GR following exposure to DEE have yielded somewhat inconclusive findings. Liu et al. [50] showed that long-term exposure of male Sprague-Dawley (SD) rats to PM2.5 (20 mg/kg for 4 weeks) from DEE decreased the SOD activity in the testes. However, Cao et al. [73] demonstrated a dose-dependent increase in the SOD activity in the testes of SD rats exposed to fine PM (10 or 20 mg/kg/day for 4 weeks). Along with the lack of changes in SOD, GPx, and GR, we found a decreased level of reduced form of glutathione (GSH) and the GSH/GSSG ratio in rats exposed to SHB20 DEE, accompanied by the increased level of oxidized form of glutathione (GSSG). This result may be interpreted as evidence of redox unbalance, which in turn may reflect the prolonged oxidative stress and impairment of antioxidant defenses due to the low ability to scavenge ROS. This conclusion is based on several observations showing that the amount of free GSH decreases when cellular systems are unable to counteract the oxidative-mediated insults [74]. Moreover, it should be kept in mind that the increased level of GSSG causes the GSH depletion what may result in diminished antioxidant defense [75]. Interestingly, we observed the increased levels of GSH and GSSG without the increase the GSH/GSSG ratio in rats exposed to B7 DEE. The significant increase of GSH level is probably a response to the increased generation of ROS. Additionally, a concomitant increase in the GSSG level may suggest de novo GSH synthesis leading to protection against oxidative stress [76]. Comparing our results to the literature, partially similar results were reported by Kisin et al. [11], who observed the reduced level of GSH in the testes of C57BL/6 mice exposed to particles from the combustion of BD50 biofuel. Another group also demonstrated the decreased GSH level and the GSH to GSSG ratio in the testes of ICR mouse exposed intragastrically to 1-nitropyrene from the DEE [77].

According to currently acceptable hypothesis, the oxidant and anti-oxidative parameters are directly interactive with pro-inflammatory markers and set up two scenarios. On the one hand, it is believed that when ROS production goes beyond their clearance, excessive or accumulated ROS can disrupt redox balance and induce oxidative stress and proinflammatory response. On the other hand, a considerable body of experimental evidence has shown that respiratory burst made by inflammatory cells during inflammation can lead to an increased production of ROS, which stimulates changes in the oxidant/antioxidant balance. We have made an attempt to clarify this issue and we have determined the testicular IL-1β, IL-6, and TNFα gene expressions and protein levels in rats exposed to DEE from B7 and SHB20 biofuels with and without DPF-treatment and with or without blackcurrant pomace supplementation (BC) for 28 days (Table S5 in Supplementary Materials). Our results revealed that only DEE from SHB20 biofuel with and without DPF-treatment increased significantly the IL-6 gene expression. Dietary intervention attenuated this effect, though the IL-6 gene expression was still significantly higher than in the control group. The expression of IL-1β and TNFα genes in the testes were not altered by DEE from B7 and SHB20 biofuels with and without DPF-treatment. In addition, no significant association between the testicular IL-1β, IL-6, and TNFα gene expressions and protein levels was observed. To sum up, the obtained results may suggest that the first scenario is more likely, i.e., that the exhaust emissions from B7 and SHB20 biodiesel blends combustion induced ROS, which in turn disrupted redox balance and induced oxidative stress and weak pro-inflammatory response, manifested as the increased IL-6 gene expression.

Recapitulating, the presented results clearly revealed that DEE from the combustion of both biofuels affected the antioxidant system and caused oxidative reactions likely triggered by ROS, which was unable to prevent oxidative reactions triggered by them. However, dietary intervention with BC significantly mitigated these redox-effects, demonstrated as the increased activities of TAS, SOD, GPx, and GR in the DEE-exposed rats. We further observed that treatment with BC significantly elevated the GSH level, decreased the concentration of GSSG, and prevented a drop in the GSH:GSSG ratio. These results are in agreement with previous studies showing that anthocyanins, mainly cyanidin-3-glucoside, exerted strong antioxidative effects not only by scavenging intracellular ROS, but also by modulating specific signaling pathway of antioxidant adaptive response [78,79]. For instance, Ye et al. [80] reported that cyanidin-3-glucoside intervention inhibited the intracellular ROS and O^2−^ levels, as well as restored the impaired activity of SOD in diabetic db/db mice. Sukprasansap et al. [81] presented the dramatically activated gene expression of SOD and GPx in cyanidin-3-glucoside-treated cells. In addition, Zhu et al. [82] shown that the cyanidin-3-*O*-glucoside lowered the oxidative stress through GSH-based antioxidant defense mechanism. Moreover, Casati et al. [83] reported that other blackcurrant anthocyanin, delphinidin-3-rutinoside, exerted its protective effect against oxidative damage by reducing intracellular ROS and increasing intracellular antioxidant factors such as GSH in MC3T3-E1 cells. Dietary intervention with BC significantly reduced the increased concentration of TBARS, 7-KCh, and LOOHs in the testes of rats exposed to DEE. These results are in line with previous studies, showing the protective effects of anthocyanins from blackcurrants against oxidative stress and other redox-related responses [83,84]. Our results showed the high concentrations of anthocyanins in blackcurrant pomace, mainly cyanidin-3-glucoside, delphinidin-3-O-glucoside, cyanidin-3-O-rutinoside, and delphinidin-3-O-rutinoside (Supplementary Materials), which possess the ROS-scavenging activity against hydroxyl radicals and superoxide, and metal-chelating abilities in vitro and in vivo [22,85,86]. The cyanidin-3-glucoside markedly decreased oxidative damage, leading to the decreased level of TBARS during serum formation in rats [87]. Similar results were reported by Nowak et al. [88], who reported that anthocyanins from blackcurrant leaves exhibited inhibitory effect on the generation of TBARS. More recently, Tan et al. [89] reported that cyanidin-3-glucoside substantially decreased the MDA levels via improving antioxidant enzymes activities (SOD, GSH-Px, and CAT) in H_2_O_2_-induced oxidative stress in HepG2 cells.

## 5. Conclusions

In conclusion, the exposure of rats to diesel exhaust emissions (DEE) from the combustion of 1st generation (B7) and 2nd generation (SHB20) biofuels without and with DPF-treatment caused oxidative stress in the testes, manifested by the increased lipid peroxidation and reduced capacity of antioxidant defense system. Our results revealed that dietary intervention with blackcurrant pomace, could attenuate these deleterious effects through a reduction in TBARS, LOOHs, 25(OH)2Ch, and 7-KCh, accompanied by an increase in TAS, SOD, GSH-Px, GR, and GSH activities. This effect of blackcurrant pomace, regarded as a good source of anthocyanins, could be related to their capacity to scavenge free radicals, and modulating specific signaling pathways of antioxidant adaptive response. Nevertheless, it must be pointed out that despite the ameliorating effect of dietary intervention with blackcurrant pomace, the redox balance in the testes was not completely restored. We reported for the first time that exposure of animals to exhaust emissions from 2nd generation biodiesel (SHB20) revealed higher markers of oxidative stress in the testes than exposure to conventional B7 biofuel. A novelty of the presented research was to investigate the effects of dietary intervention with blackcurrant pomace that is a residue material obtained during juice production in animals exposed to DEE at a level relevant to human exposure in urban agglomerations. Cars equipped with diesel engines are still very common especially in many European countries and contribute to urban air pollution. Considering the fact that traffic-related air pollution negatively affects male reproductive potential and reduction of the impact of diesel cars on environment is a long-term effort in many countries, there is a need to implement alternative methods diminishing negative health effects from the exposition to DEE. According to our opinion, a dietary intervention with compounds presenting a high antioxidative potential can be a solution to improve a redox balance in the reproductive system.

## Data Availability

The data are contained within the article and supplementary materials.

## Supplementary Materials

The following supporting information can be downloaded at: https://www.mdpi.com/article/10.3390/antiox11081562/s1, Figure S1: Average weekly feed intake by rats exposed to DEE from B7 or SHB20 biofuels without (A) or with (B) DPF-treatment and with or without blackcurrant pomace supplementation (BC) for 28 days; Figure S2: The scheme of the exposure of the test fuels to the animals; Table S1: Chemical analysis of experimental feeds composition [90,91]; Table S2: The content of selected phenolics in experimental feeds [92,93]; Table S3. The estimated phenolics mean daily intake in rats exposed to B7 and SHB20 diesel fuel exhaust emission with (DPF+) or without (DPF-) diesel particle filter (DPF) application (mean ± SE); Table S4. RT-PCR primer sequences of analyzed genes; Table S5. Testicular IL-1β, IL-6, and TNFα gene expressions and protein levels in rats exposed to DEE from B7 and SHB20 biofuels with and without DPF-treatment and with or without blackcurrant pomace supplementation (BC) for 28 days; Table S6. Estimated concentration of selected substances in the whole body inhalation chambers (mean ± SD); Table S7. The estimated concentration of the selected aromatic hydrocarbons in the whole body inhalation chambers (mean, ± SD).
