# Supplementary material for: Dietary Intervention with Blackcurrant Pomace Protects Rats from Testicular Oxidative Stress Induced by Exposition to Biodiesel Exhaust
Source: Antioxidants (Basel). 2022 Aug 12;11(8):1562. doi: 10.3390/antiox11081562 (PMC9404818; doi:10.3390/antiox11081562)
Supplement: Supplementary file 1 [file antioxidants-11-01562-s001.zip › antioxidants-1811916-supplementary.pdf]

Dietary intervention with blackcurrant pomace protects rats from testicular oxidative stress induced by exposition to biodiesel exhaust

Michał Oczkowski [1,*], Jacek Wilczak [2], Katarzyna Dziendzikowska [1], Johan Øvrevik [3,4], Oddvar Myhre [3], Anna Lankoff [5,6], Marcin Kruszewski [5,7] and Joanna Gromadzka-Ostrowska [1]

[1] Department of Dietetics, Institute of Human Nutrition Sciences, Warsaw University of Life Sciences (WULS-SGGW), 159c Nowoursynowska Str., 02-776 Warsaw, Poland
[2] Department of Physiological Sciences, Institute of Veterinary Medicine, Warsaw University of Life Sciences (WULS-SGGW), 159 Nowoursynowska Str., 02-776 Warsaw, Poland
[3] Department of Environmental Health, Norwegian Institute of Public Health, Lovisenberggata 8 Str., 0456 Oslo, Norway
[4] Department of Biosciences, Faculty of Mathematics and Natural Sciences, University of Oslo, Blindernveien 31 Str., 0371 Oslo, Norway
[5] Centre for Radiobiology and Biological Dosimetry, Institute of Nuclear Chemistry and Technology, 16 Dorodna Str., 03-195 Warsaw, Poland
[6] Division of Medical Biology, Institute of Biology, Jan Kochanowski University, 7 Uniwersytecka Str., 25-406 Kielce, Poland
[7] Department of Molecular Biology and Translational Research, Institute of Rural Health, 7 Jaczewskiego Str., 20-090 Lublin, Poland
* Correspondence: michal_oczkowski@sggw.edu.pl; Tel.: +48-225937031

Supplementary material

List of sections

S1. Reagents and chemicals ………………………………………………..…………….……..….. **page 2**

S2. Chemical analysis of experimental feeds composition ………………………………………..…. **page 3**

S3. Analysis of selected flavonoids and phenolic acids in feed supplemented with blackcurrant pomace ……………………………………………………………………………………….........…. **page 4**

S4. The estimated mean daily intake of selected phenolic compounds …………………………… **page 6**

S5. Feed intake in rats exposed to DEE from B7 or SHB20 biofuels ……….…………………….. **page 7**

S6. Testicular IL-1β, IL-6, and TNFα gene expressions and protein levels in rats exposed to DEE from B7 and SHB20 biofuels ………………………………………………………….…………….…… **page 8**

S6.1. RNA isolation, reverse transcription, and real-time PCR ……………….……….... **page 8**

S6.2. Immunoenzymatic (ELISA) determination of cytokine concentrations (IL-1β, IL-6, TNFα) in testis ……………………………………………………………………………………… **page 8**

S7. Estimated concentration of selected compounds in the whole body inhalation chambers .... **page 10**

S8. The scheme of the exposure of the test fuels to the animals …………………………………… **page 12**

S1. Reagents and chemicals

The reagents and chemicals were purchased from Sigma-Aldrich (St. Louis, MO, USA), unless otherwise indicated.

Isoflurane (*Aerrane Isofluranum*) for animals anesthesia was purchased from Baxter® (Deerfield, USA). Phosphate buffered saline (PBS), potassium dihydrogen phosphate (KH_2PO_4; AVANTOR™ Performance Materials, formerly POCH, Gliwice, Poland), di-potassium hydrogen phosphate (K_2HPO_4), ethylenediaminetetraacetic acid (EDTA; AVANTOR™ Performance Materials, formerly POCH, Gliwice, Poland) were used for testis samples preparation for analysis of oxidative stress parameters and antioxidative defense markers. Thiobarbituric acid (TBA), sulfuric acid (H_2SO_4) min. 95% (AVANTOR™ Performance Materials, formerly POCH, Gliwice, Poland), n-butanol, 1,1,3,3-tetramethoxypropane were used for determination of lipid peroxidation determined as concentration of substances reacting with thiobarbituric acid (TBARS). 3-(N-Morpholino)propanesulfonic acid sodium salt (MOPS), Triton X-100, lipoprotein lipase, trimethylenetetraminehexaacetic acid, ascorbate oxidase, 2-methylbenzimidazole, sarcosinate LN, cumene hydroperoxide (as external standard) were used for lipid hydroperoxides determination. Methanol, hexane, butylated hydroxyanizol, acetonitrile (J.T. Baker, Phillipsburg, USA), ammonium acetate and dithiothreitol were used for detection of 25-dihydroxycholesterol and 7-ketocholesterol. Monobasic sodium phosphate, 1-octanesulfonic acid, acetonitrile (J.T. Baker, Phillipsburg, USA), *meta*-phosphoric acid were used for determination of reduced and oxidized forms of glutathione. Acetic acid, acetonitrile (J.T. Baker, Phillipsburg, USA), methanol, standards of gallic acid, chlorogenic acid, myricetin, quercetin, kaempferol, quercetin-3-*O*-rutinoside, quercetin-3-*O*-glucoside cyanidin-3.5-di-*O*-rutinoside, delphinidin-3.5-di-*O*-rutinoside, delphinidin-3.5-di-*O*-glucoside and cyanidin-3.5-di-O-glucoside were used as standards for detection of flavonoids and phenolic acids in feed. QIAzol reagent (Qiagen, Hilden, Germany), nuclease-free water, TRIS-base, HCl (AVANTOR™ Performance Materials, formerly POCH, Gliwice, Poland), EDTA were used to pro-inflammatory genes expression analysis. PBS, protease inhibitor cocktail (Merck, Darmstadt, Germany), EDTA, Igepal, Tween 20, sodium dodecyl sulfate (SDS) were used for preparation of testis homogenates for analysis of proteins of proinflammatory cytokines.

S2. Chemical analysis of experimental feeds composition

The chemical analysis of experimental feeds composition was performed at certified laboratory (Merieux NutriScience Silliker, Warsaw, Poland). The content of water, dry matter, crude nitrogen, crude protein, crude fat, and ash was determined according to methods recommended by the Commission Regulation (EC) No 152/2009 [90]. Crude fiber analysis was performed according to method described in Polish Standard PN-EN ISO 6865:2002 [91]. The content of carbohydrates was calculated by subtracting content of water, fat, protein and crude ash from 100g.

Table S1. Chemical analysis of experimental feeds composition

Component	Feed supplemented with blackcurrant pomace	Feed without supplementation
water (g/100g)	13.0±0.2	14.1±0.2
dry matter (g/100g)	87.0	85.9
crude nitrogen	2.63±0.37%	2.58±0.36%
crude protein (g/100 g)	16.4±2.3	16.1±2.3
crude fat (%)	5.9±1.8	5.5±1.7
carbohydrates (g/100 g)	62.0	61.7
crude ash (%)	2.63±0.37	2.57±0.36
crude fiber (%)	4.1±0.4	3.7±0.4

References

90. Commission Regulation (EC) No 152/2009 of 27 January 2009 laying down the methods of sampling and analysis for the official control of feed (Text with EEA relevance). Official Journal of European Union, L54/1
91. PN-EN ISO 6865:2002. Animal feeding stuffs — Determination of crude fibre content - Method with intermediate filtration. Polish Committee of Standardization, Warsaw, Poland

S3. Analysis of selected flavonoids and phenolic acids in feed supplemented with blackcurrant pomace

Analysis of selected phenolic (gallic and chlorogenic), flavonols (myricetin, quercetin, kaempferol, quercetin-3-*O*-rutinoside, quercetin-3-*O*-glucoside), and anthocyanidins (cyanidin-3.5-di-*O*-rutinoside, delphinidin-3.5-di-*O*-rutinoside, delphinidin-3.5-di-*O*-glucoside, cyanidin-3.5-di-*O*-glucoside) in feed with blackcurrant pomace was performed according to the method described by Hallmann et al. [92]. The mobile phase was acetonitrile 10% and 55% mixed with ultrapure deionized water. Detection of phenolic acids and flavonoids was made at the wavelength of 280 nm and 340 nm, respectively. Polyphenols were assessed in 3 replicates and 5 injections of external standards (purity of 99.0 %) were prepared.

Analysis of anthocyanins were performed according to method described by Dóka et al. [93]. The elution of anthocyanins from the extract (1 mL) was made using isocratic flow with mobile phase (a mixture of 5% acetic acid, acetonitrile and methanol, 70:10:20 v/v/v) with a flow rate of 1.5 mL/min. Detection of anthocyanins was performed at 530 nm. Individual compounds were identified based on pure standards (purity of 99.9%) and retention times from chromatograms.

Table S2. The content of selected phenolics in experimental feeds

class of phenolic compound	compound (mg/100 g of feed)	feed supplemented with blackcurrant pomace	feed without blackcurrant pomace
phenolic acids	gallic acid	0.56±0.00	N.D.[#]
	chlorogenic acid	0.71±0.02	N.D.
flavonols	myricetin	0.58±0.01	N.D.
	quercetin	0.27±0.00	N.D.
	kaempferol	0.04±0.01	N.D.
	quercetin-3-*O*-rutinoside	2.41±0.01	N.D.
	quercetin-3-*O*-glucoside	1.08±0.01	N.D.
anthocyanidins	cyanidin-3.5-di-*O*-rutinoside	20.28±0.11	N.D.
	delphinidin-3.5-di-*O*-rutinoside	27.51±0.12	N.D.
	delphinidin-3.5-di-*O*-glucoside	8.26±0.03	N.D.
	cyanidin-3.5-di-*O*-glucoside	18.42±0.02	N.D.

[#] N.D. – not detected

References

92. Hallmann, E.; Kazimierczak, R.; Marszałek, K.; Drela, N.; Kiernozek, E.; Toomik, P.; Matt, D.; Luik, A.; Rembiałkowska, E. The Nutritive Value of Organic and Conventional White Cabbage (Brassica Oleracea L. Var. Capitata) and Anti-Apoptotic Activity in Gastric Adenocarcinoma Cells of Sauerkraut Juice

Produced Therof. Journal of Agricultural and Food Chemistry 2017, 65, 37, 8171-8183, doi:10.1021/acs.jafc.7b01078.
93. Dóka, O.; Ficzek, G.; Bicanic, D.; Spruijt, R.; Luterotti, S.; Tóth, M.; Buijnsters, J.G.; Végvári, G. Direct photothermal techniques for rapid quantification of total anthocyanin content in sour cherry cultivars. *Talanta* 2011, 84, 2, 341-346, doi:10.1007/s10967-012-2320-y.

S4. The estimated mean daily intake of selected phenolic compounds

Table S3. The estimated mean daily intake of phenolic compounds in rats exposed to B7 and SHB20 diesel fuel exhaust emission with (DPF+) or without (DPF-) diesel particle filter (DPF) application (mean ± SE).

Phenolic compound intake (mg/d)	rats from experimental groups			
	with DPF-treatment		without DPF-treatment	
	B7+BC (n=6)	SHB20+BC (n=6)	SHB20+BC (n=6)	SHB20+BC (n=6)
gallic acid	0.085±0.001	0.078±0.002*	0.078±0.002*	0.073±0.002**
chlorogenic acid	0.108±0.001	0.099±0.003*	0.099±0.003*	0.093±0.002**
myricetin	0.088±0.001	0.081±0.002*	0.081±0.002*	0.076±0.002**
quercetin	0.041±0.001	0.038±0.001*	0.038±0.001*	0.035±0.001**
kaempferol	0.006±0.000	0.006±0.000*	0.006±0.000*	0.005±0.0001**
quercetin-3-*O*-rutinoside	0.333±0.034	0.336±0.009	0.336±0.009	0.315±0.009
quercetin-3-*O*-glucoside	0.165±0.001	0.151±0.004*	0.151±0.004*	0.141±0.004**
cyanidin-3.5-di-*O*-rutinoside	3.091±0.028	2.831±0.078*	2.831±0.078*	2.649±0.076**
delphinidin-3.5-di-*O*-rutinoside	4.192±0.038	3.840±0.105*	3.840±0.105*	3.593±0.103**
delphinidin-3.5-di-*O*-glucoside	1.259±0.011	1.153±0.032*	1.153±0.032*	1.079±0.031**
cyanidin-3.5-di-*O*-glucoside	2.807±0.026	2.571±0.071*	2.571±0.071*	2.406±0.069**

* *vs.* B7+BC (without DPF) group, $P<0.05$; ** *vs.* B7+BC (with DPF) group, $P<0.05$ (T-Student test; analysis conducted separately for with or without DPF-treatment)

S5. Feed intake in rats exposed to DEE from B7 or SHB20 biofuels

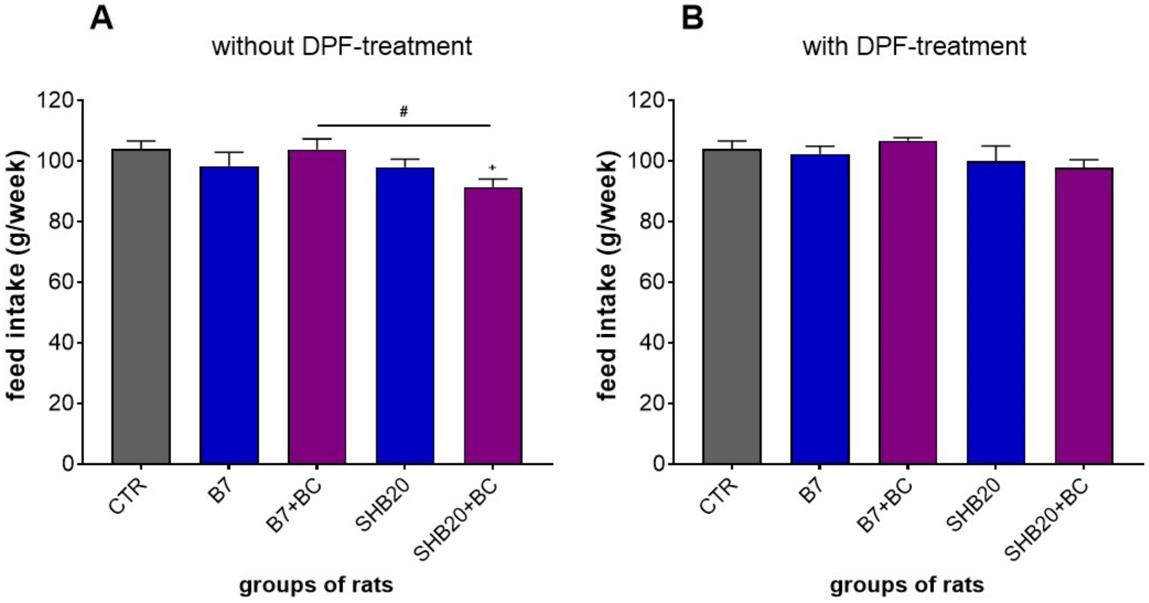

Figure S1. Average weekly feed intake in rats exposed to DEE from B7 or SHB20 biofuels without (A) or with (B) DPF-treatment and with or without blackcurrant pomace supplementation (BC) for 28 days. CTR – control group; B7 - 1st generation biofuel; SHB20 – 2nd generation biofuel; BC – blackcurrant pomace; DPF – diesel particulate filter. Data are expressed as mean ± SEM. # - significant difference between normal and blackcurrant pomace supplemented diet, # *P*<0.05; + - Statistically significant difference from the control group (CTR), + *P*<0.05, one-way ANOVA with Duncan's *post-hoc* test.

S6. Testicular IL-1β, IL-6, and TNFα gene expressions and protein levels in rats exposed to DEE from B7 and SHB20 biofuels

S6.1. RNA isolation, reverse transcription, and real-time PCR

RNA isolation and reverse transcription

Testis samples (50-100 mg) were homogenized in 1 mL of QIAzol reagent (Qiagen, Hilden, Germany) using a homogenizer (TissueLyser LT, Qiagen). RNA was extracted using RNeasy Lipid Tissue Mini kits (Qiagen, Hilden, Germany) according to the manufacturer's protocol. RNA was eluted in 35 μL and stored at -80 °C. To assess the concentration and purity of RNA, the portion of every RNA sample was diluted in TE buffer (pH 8.0) and the absorbance at 230, 260, and 280 nm was measured using NanoDrop 2000 spectrophotometer (NanoDrop). All RNA samples had A260/A280 and A260/A230 ratios ≥ 2.0. For PCR array analysis, total RNA was converted to complementary DNA (cDNA) using SensiFAST cDNA Synthesis Kit (Bioline LTD, UK) according to the manufacturer's protocol. Samples were subsequently diluted in nuclease-free water to final concertation 100 ng/μL and stored at -80°C.

Real-time PCR

The expression of genes was analyzed by RT qPCR using S SensiFAST™ SYBR Lo-ROX Kit using Stratagen Mx3005P instrument (Agilent). All reactions were performed in 10 μL reactions in triplicate. Negative controls were included for each gene target under assay. For each well 1 μL of cDNA from each sample was added to 9 μL of PCR reaction mix, which consisted of 5 μL SensiFAST SYBR Lo-ROX Mix master mix, 2 μL nuclease free water and 2 μL primers mix (IBB PAN, Warsaw, Poland). PCR primer sequences are shown in Table 2.

Table S4. RT-PCR primer sequences of analyzed genes

Gen name	Code	Forward	Reverse	Type
interleukin 1beta	*Il-1b*	CACCTTCTTTTCCTTC ATCTTTG	GTCGTTGCTTGTCTCTC CTTGTA	target
interleukin 6	*Il-6*	GAAATACAAAGAAA TGATGG	GTGTTTCAACATTCAT ATTGC	target
TNF alpha	*Tnfa*	ACTGAACTTCGGGGT GATCG	GCTTGGTGGTTTGCTA CGAC	target
beta-actin	*Actb*	CCTGGGTATGGAATC CTGTG	CTTCTGCATCCTGTCA GCAA	reference

PCR amplification was carried out with an initial 3-min step at 95 °C followed by 40 cycles at 95 °C for 5 seconds, 60 °C for 10 seconds and 72°C for 5 seconds, with melting curves analysis, according to the manufacturer's recommendations. Relative gene expression was calculated using the ΔΔCt method with beta-actin as reference control. Calculations were done using the GenEx ver 6 software (MultiD). Data were analyzed by n-norm test and normalized.

S6.2. Immunoenzymatic (ELISA) determination of cytokine concentrations (IL-1β, IL-6, TNFα) in testis

Frozen samples of testis were homogenized (1:2, w:v) in 0.005 M PBS buffer (pH 7.5) containing protease inhibitor cocktail (Merck, Darmstadt, Germany) and 0.001 M EDTA (for IL-1β analysis) or were homogenized (1:3, w/v) in 0.005 M phosphate buffered saline buffer (PBS, Sigma-Aldrich, St Louis, USA) containing 1% Igepal (Sigma-Aldrich, St Louis, USA), 0.5% Tween 20, 0.1% SDS (Sigma-Aldrich, St Louis, USA) and protease inhibitor cocktail (Merck, Darmstadt, Germany) (pH 7.5) (O'Bryan et al.,

2005) (for IL-6 and TNFα determination). After centrifugation at 18 000xg (30 minutes at 4C°), the supernatants were stored at -80°C until further use. The protein level of IL-1β was determined by using Quantikine® ELISA kit for rats (R&D Systems, Minneapolis, USA; cat. no RLB00) according to the manufacturer's protocol. The intra- and inter-assay coefficients of variation were ≤ 8.8% and ≤ 5.7%, respectively. The minimum detectable dose of IL-1β was 5 pg/mL. The protein level of IL-6 was determined by using Quantikine® ELISA kit for rats (R&D Systems, Minneapolis, USA; cat. no R6000B) according to the manufacturer's protocol. The intra- and inter-assay coefficients of variation were ≤ 8.8% and ≤ 10.0%, respectively. The minimum detectable dose of IL-6 was 21 pg/mL. The protein level of TNFα was analyzed by using Quantikine® ELISA kit for rat (R&D Systems, Minneapolis, USA; cat. no RTA00) according to the manufacturer's protocol. The intra- and inter-assay coefficients of variation were ≤ 5.1% and ≤ 9.7%, respectively. The sensitivity of the assay was less than 5 pg/mL. Cytokine levels in tissue homogenates were calculated per mg of protein determined by using Pierce BCA Protein Assay Kit (Thermo Scientific, Waltham, USA) according to the manufacturer's protocol.

Table S5. Testicular IL-1β, IL-6, and TNFα gene expressions and protein levels in rats exposed to DEE from B7 and SHB20 biofuels with and without DPF-treatment and with or without blackcurrant pomace supplementation (BC) for 28 days.

	CTR group	Experimental groups of rats without DPF-treatment (DPF-)				Experimental groups of rats with DPF-treatment (DPF+)			
		B7	B7+BC	SHB20	SHB20+BC	B7	B7+BC	SHB20	SHB20+BC
gene expression (relative mRNA expression):									
Il-1b	1.00±0.10	0.94±0.09	0.92±0.11	1.13±0.04	0.95±0.15	1.10±0.09	1.31±0.11	1.20±0.08	1.06±0.06
Il-6	1.00±0.18	1.25±0.29^{A}	1.25±0.20	3.23±0.42^{+++}	1.98±0.27^{B+}	2.00±0.37	0.87±0.08	3.51±0.39^{++}	2.80±0.82^{+}
Tnfa	1.00±0.08	1.09±0.16	1.14±0.11	1.07±0.09	1.11±0.17	1.13±0.14	1.33±0.17	1.43±0.31	1.17±0.12
cytokine protein level (pg/mg protein):									
IL-1β	2.37±0.33	3.11±0.32^{CD}	1.54±0.31	1.63±0.19	1.48±0.29	2.33±0.31	1.87±0.14	2.06±0.28	1.50±0.45
IL-6	5.53±0.56	5.29±0.62	4.78±0.19	5.52±0.91	4.69±0.50	4.83±0.88	6.33±1.02	6.18±0.85	6.36±0.54
TNFα	0.36±0.04	0.45±0.62	0.40±0.07	0.35±0.03	0.44±0.08	0.37±0.05	0.40±0.06	0.44±0.06	0.50±0.05

Data are expressed as mean ± SEM. A – significantly different *vs.* SHB20 in DPF(-) group (P<0.001); B – significantly different *vs.* SHB20 in DPF(-) group (P<0.05); C – significantly different *vs.* SHB20 group (P<0.01); D – significantly different *vs.* B7+BC group (P<0.001); +, ++, +++ - *vs.* CTR group (P<0.05, P<0.01, P<0.001), one-way ANOVA with Duncan's *post-hoc* test.

S7. Estimated concentration of selected compounds in the whole body inhalation chambers*

Table S6. Estimated concentration of selected substances in the whole body inhalation chambers (mean, ± SD)

compound	B7 diesel blend		SHB20 diesel blend	
	with DPF-treatment	without DPF-treatment	with DPF-treatment	without DPF-treatment
carbon dioxide [g/m^3]	3.6	3.60	3.60	3.60
exhaust dilution ratio [%] (set value)	2.14±0.003	2.17±0.075	2.19±0.025	2.26±0.078
carbon dioxide [g/m^3]	3.62±0.19	3.63±0.22	3.62±0.19	3.63±0.22
carbon monoxide [mg/m^3]	LoQ	2.60±0.16	LoQ	2.71±0.17
hydrocarbons [mg/m^3]	0.901±0.046	0.798±0.048	1.07±0.055	0.550±0.033
NOx [mg/m^3]	38.0±2.0	41.3±2.5	38.4±2.0	41.9±2.55
particulate matter [mg/m^3]	0.17±0.01	2.19±0.13	0.19±0.01	2.13±0.13

LoQ - Below the limit of quantification

Table S7. The estimated concentration of the selected aromatic hydrocarbons in the whole body inhalation chambers (mean, ± SD)

compound	B7 diesel blend		SHB20 diesel blend	
	with DPF-treatment	without DPF-treatment	with DPF-treatment	without DPF-treatment
Naphthalene [pg/m^3]	10.47 ± 0.54	892.11 ± 54.19	4.49 ± 0.23	692.12 ± 42.04
Acenaphthylene [pg/m^3]	0.32 ± 0.02	59.36 ± 3.6	2.67 ± 0.14	29.96 ± 1.82
Acenaphthalene [pg/m^3]	0.89 ± 0.05	25.32 ± 3.61	0.93 ± 0.05	15.90 ± 0.97
Fluorene [pg/m^3]	0.71 ± 0.04	128.20 ± 7.79	0.37 ± 0.02	51.91 ± 3.15
Phenanthrene [pg/m^3]	1.38 ± 0.07	2 848.41 ± 173.03	0.93 ± 0.05	1 135.51 ± 68.98
Anthracene [pg/m^3]	LoQ	261.66 ± 15.89	0.06 ± 0.00a	124.73 ± 7.58
Fluoranthene [pg/m^3]	0.15 ± 0.01	8 323.97 ± 505.63	0.22 ± 0.01	1 409.47 ± 85.62
Pyrene [pg/m^3]	0.29 ± 0.01	20 862.6 ± 1 267.2	0.44 ± 0.02	8 975.9 ± 545.2
Benzo(a)anthracene [pg/m^3]	1.47 ± 0.08	572.49 ± 34.78	8.85 ± 0.45	389.32 ± 23.65
Chrysene [pg/m^3]	1.95 ± 0.10	2370.75 ± 144.01	11.22 ± 0.58	1 532.03 ± 93.06
Benzo(b)fluoranthene [pg/m^3]	5.06 ± 0.26	993.96 ± 60.38	9.48 ± 0.49	620.02 ± 37.66
Benzo(k)fluoranthene [pg/m^3]	4.29 ± 0.22	597.08 ± 36.27	7.34 ± 0.38	305.33 ± 18.55
Benzo(a)pyrene [pg/m^3]	1.99 ± 0.10	87.81 ± 5.33	2.52 ± 0.13	49.39 ± 3.00
Benzo(a)fluoranthene [pg/m^3]	1.99 ± 0.10	150.32 ± 9.13	2.50 ± 0.13	59.48 ± 3.61
Indeno(1,2,3-c,d)pyrene [pg/m^3]	3.51 ± 0.18	83.94 ± 5.10	4.19 ± 0.21	692.12 ± 42.04
Dibenzo(a,h)anthracene [pg/m^3]	0.86 ± 0.04	15.10 ± 0.92	1.43 ± 0.07	29.96 ± 1.82
Benzo(g,h,i)perylene [pg/m^3]	2.50 ± 0.13	109.23 ± 6.64	3.71 ± 0.19	15.90 ± 0.97

LoQ - Below the limit of quantification

* based on published papers:

Ref. 61: Czarnocka, J.; Odziemkowska, M. Characterization of the Polycyclic Aromatic Hydrocarbons Emitted from a Compression Ignition Engine Powered with Biofuels of the 1st and 2nd Generation. *Chemik* **2016**, 70, 419–425.

Ref. 23: Dziendzikowska, K.; Gajewska, M.; Wilczak, J.; Mruk, R.; Oczkowski, M.; Żyła, E.; Królikowski, T.; Stachoń, M.; Øvrevik, J.; Myhre, O.; et al. The Effects of 1st and 2nd Generation

Biodiesel Exhaust Exposure on Hematological and Biochemical Blood Indices of Fisher344 Male Rats—The FuelHealth Project. *Environ. Toxicol. Pharmacol*. **2018**, 63, 34–47. https://doi.org/10.1016/J.ETAP.2018.08.001.

Ref. 62: Lankoff, A.; Brzoska, K.; Czarnocka, J.; Kowalska, M.; Lisowska, H.; Mruk, R.; Øvrevik, J.; Wegierek-Ciuk, A.; Zuberek, M.; Kruszewski, M. A Comparative Analysis of in Vitro Toxicity of Diesel Exhaust Particles from Combustion of 1st- and 2nd-Generation Biodiesel Fuels in Relation to Their Physicochemical Properties—The FuelHealth Project. *Environ. Sci. Pollut. Res*. **2017**, 24, 19357–19374. https://doi.org/10.1007/s11356-017-9561-9.

Ref. 26 Odziemkowska, M.; Czarnocka, J.; Frankiewicz, A.; Szewczyńska, M.; Lankoff, A.; Gromadzka-Ostrowska, J.; Mruk, R. Chemical Characterization of Exhaust Gases from Compression Ignition Engine Fuelled with Various Biofuels. *Pol. J. Environ. Stud*. **2017**, 26, 1183–1190. https://doi.org/10.15244/pjoes/67973

S8. The scheme of the exposure of the test fuels to the animals

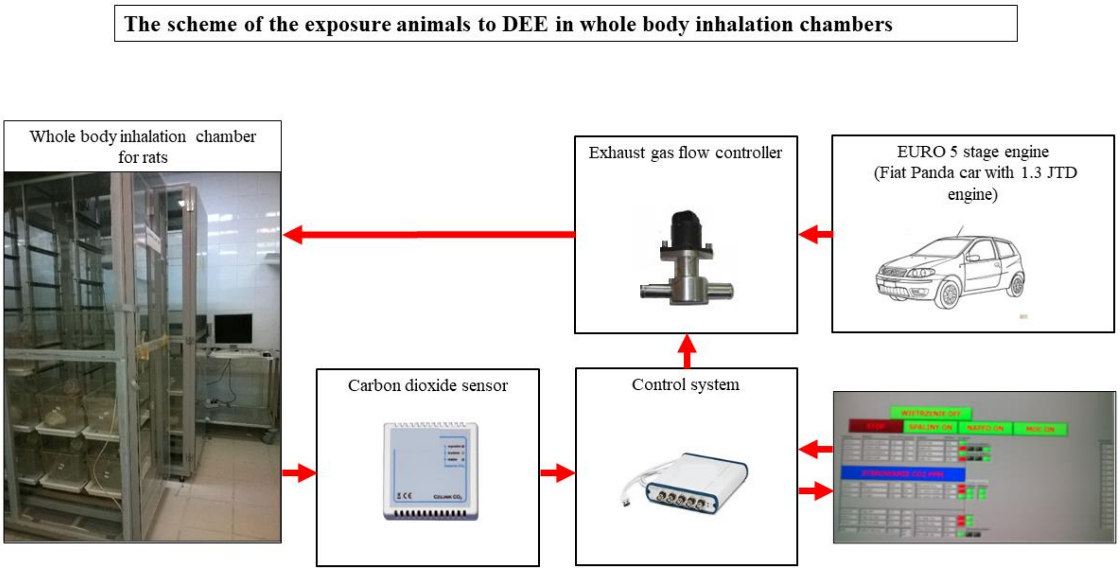

Figure S2. The scheme of the exposure of the test fuels to the animals.
